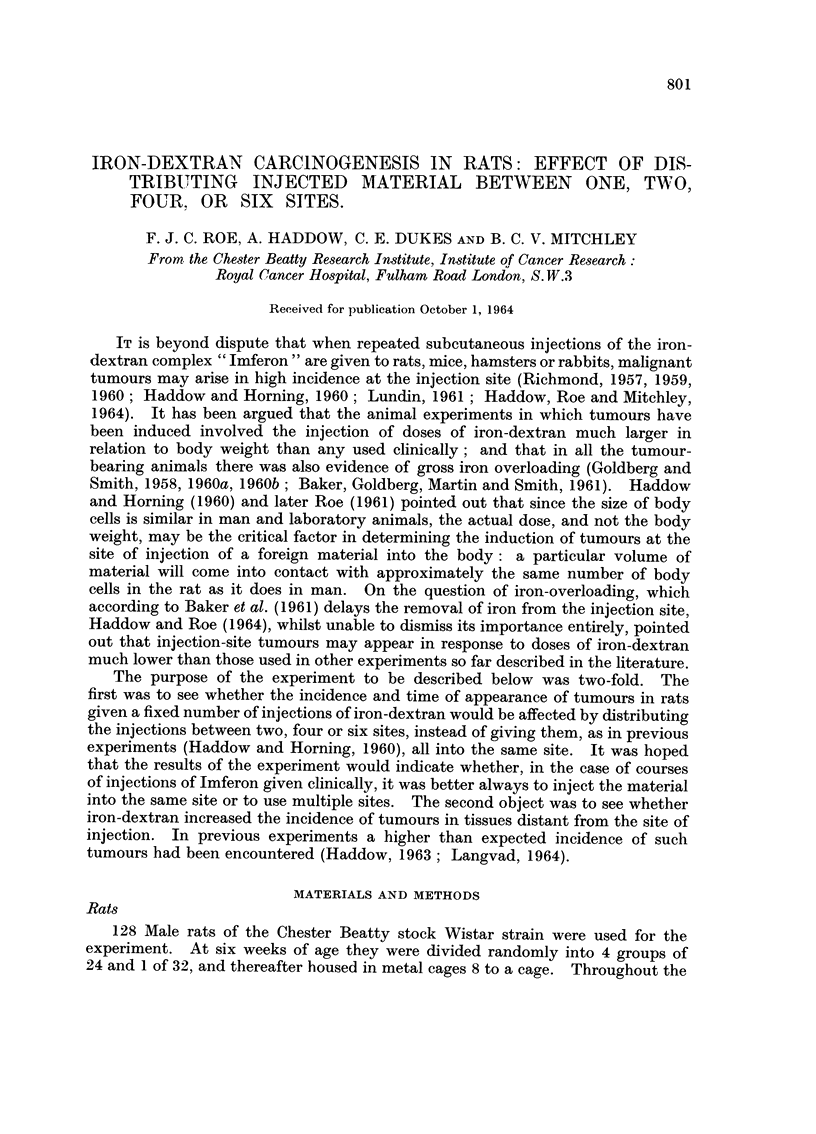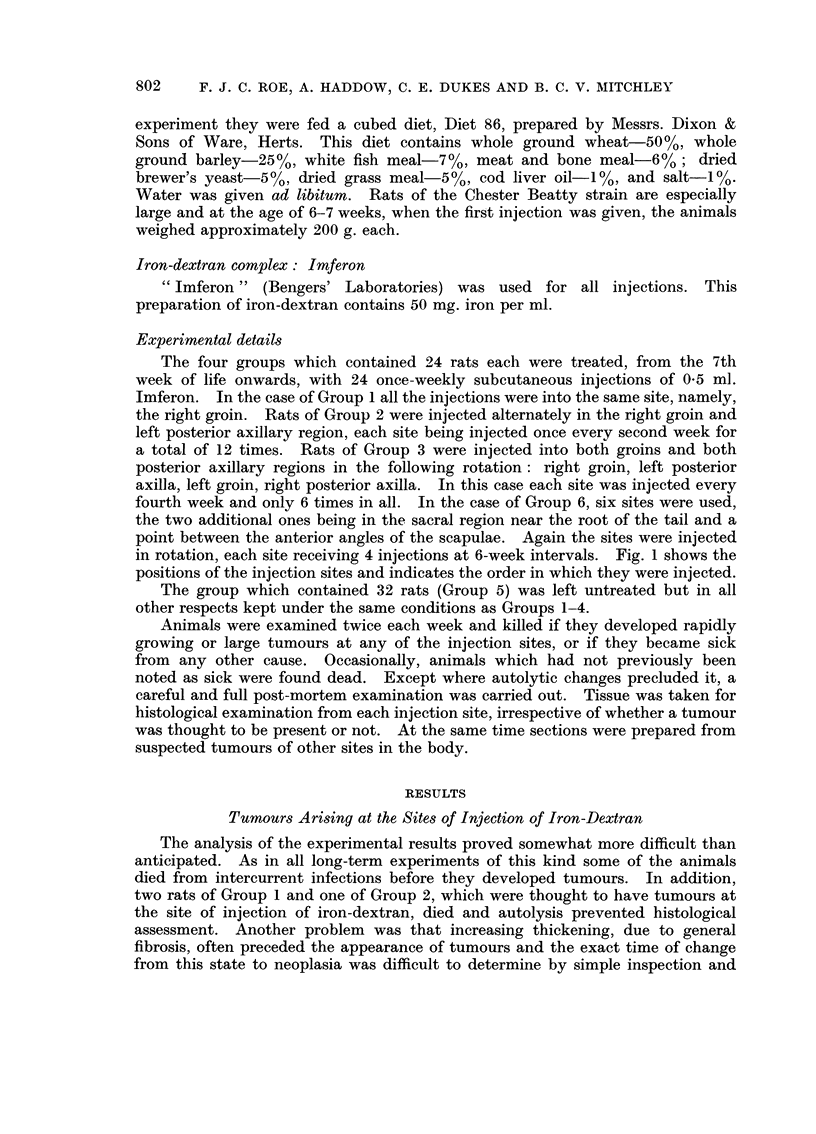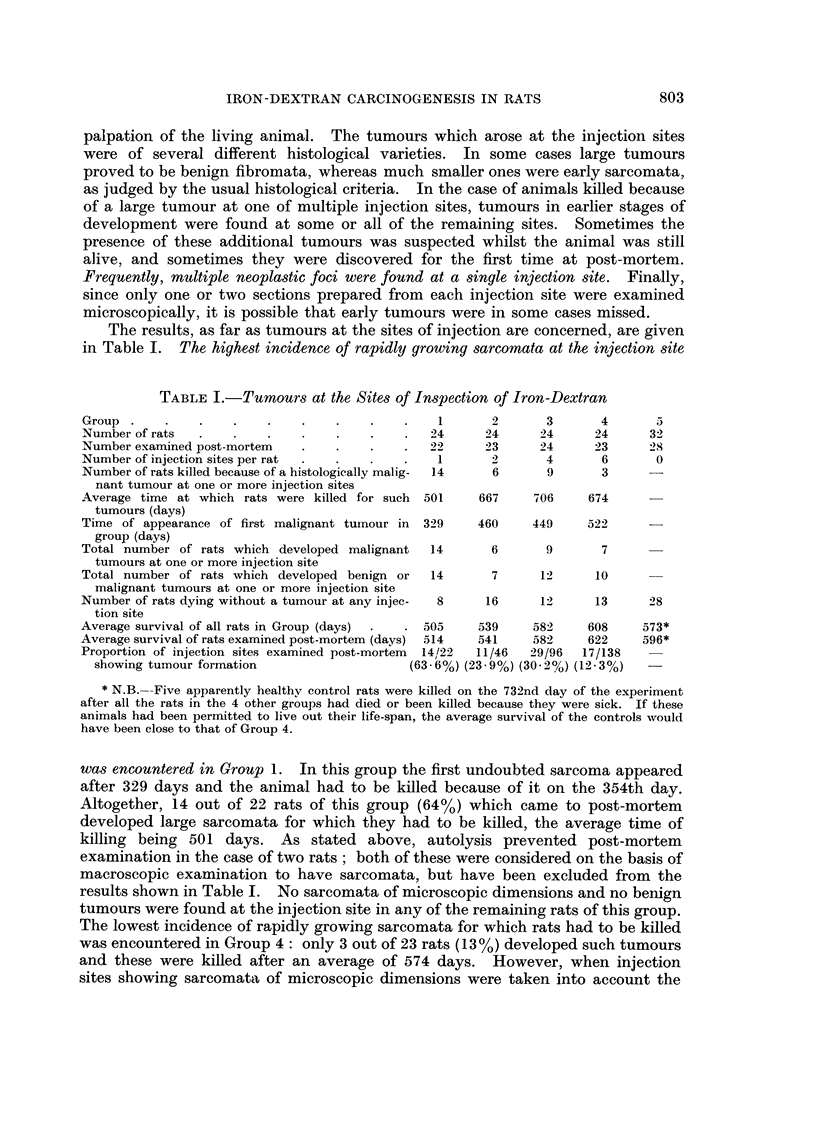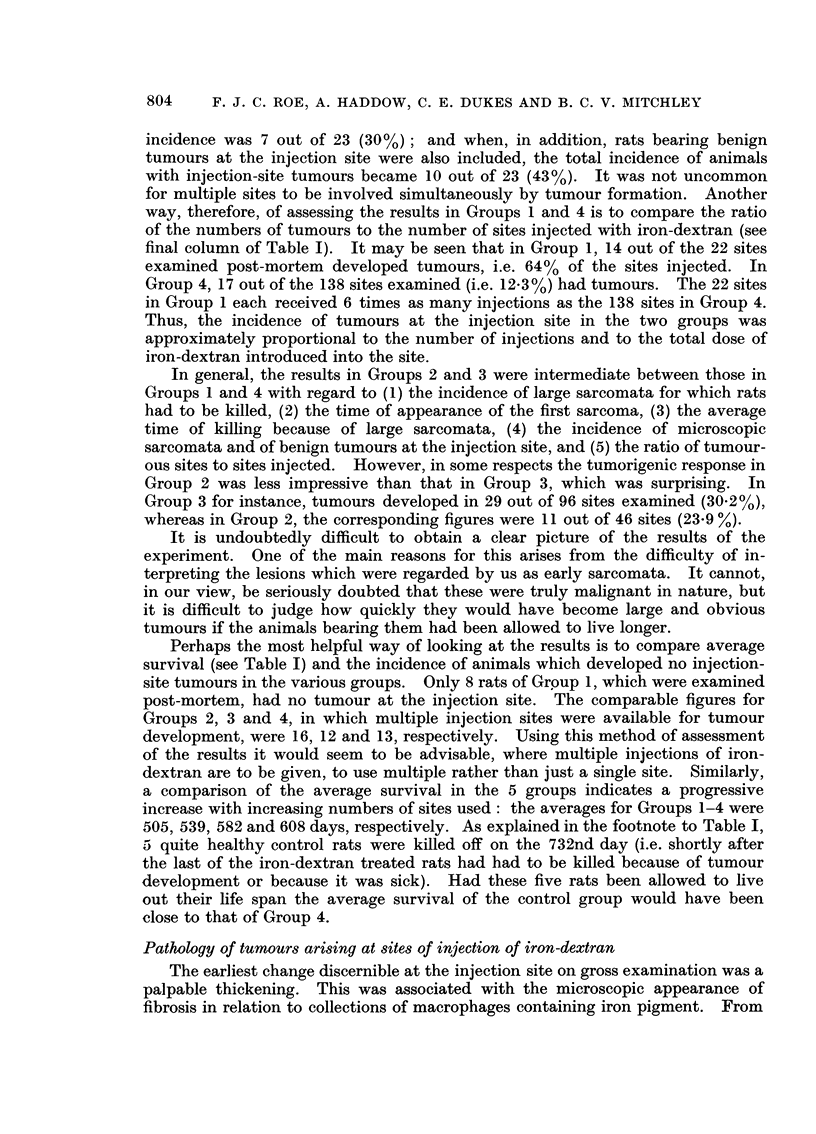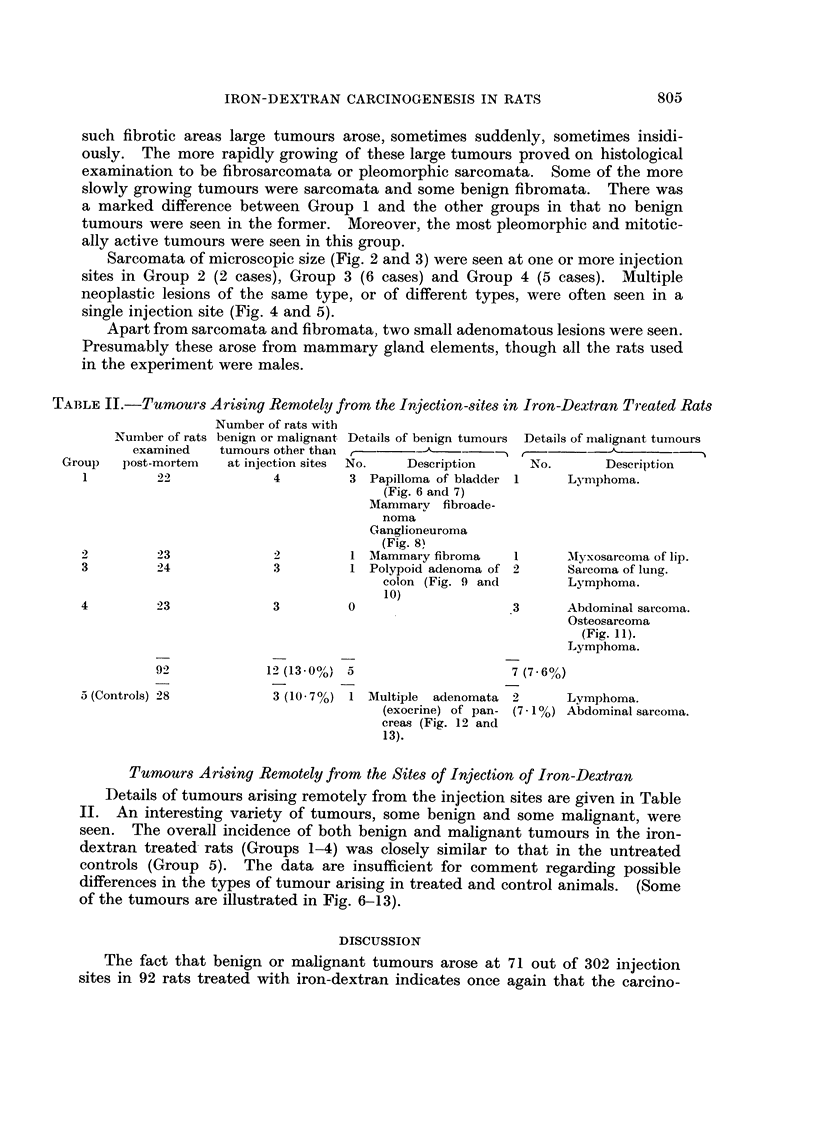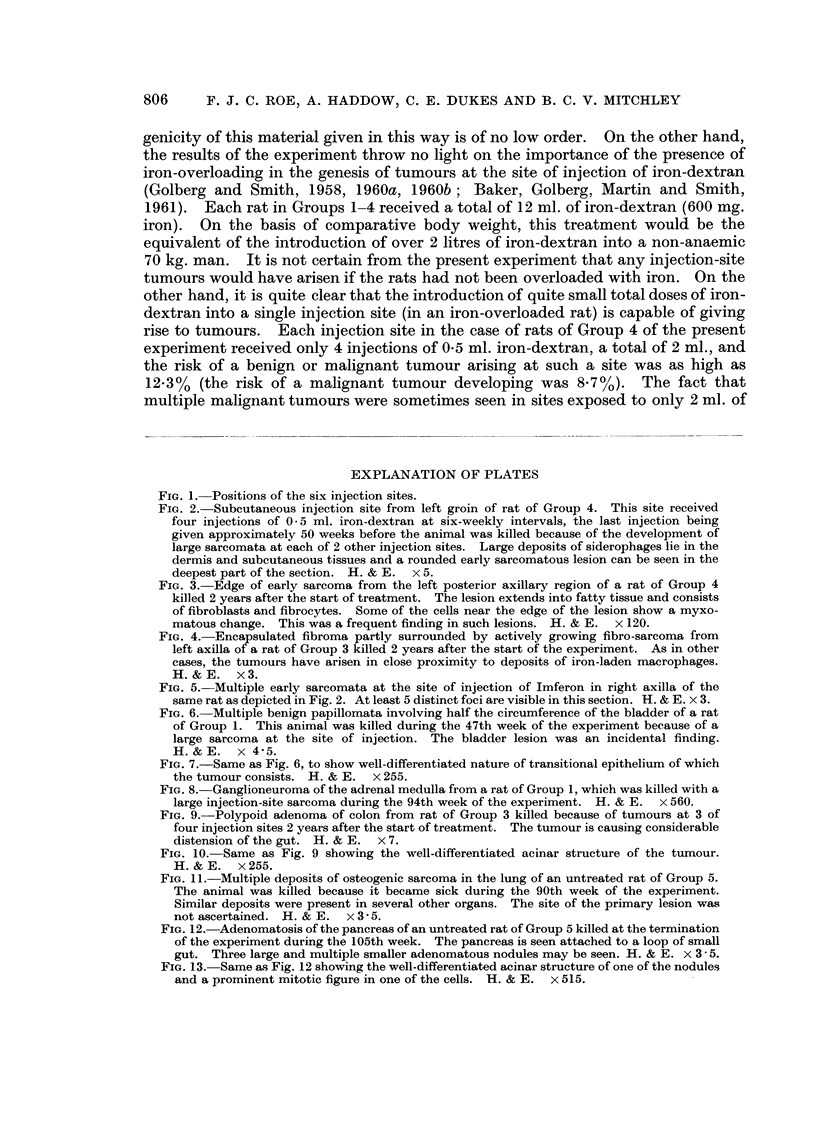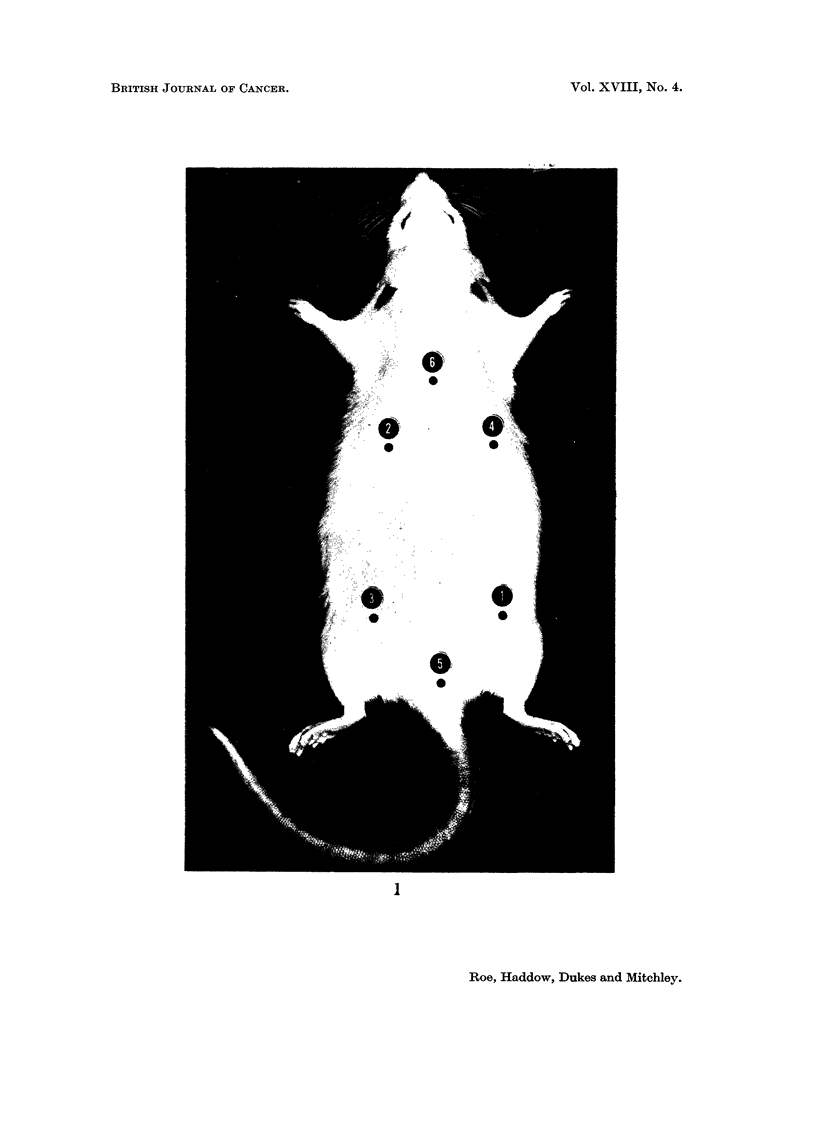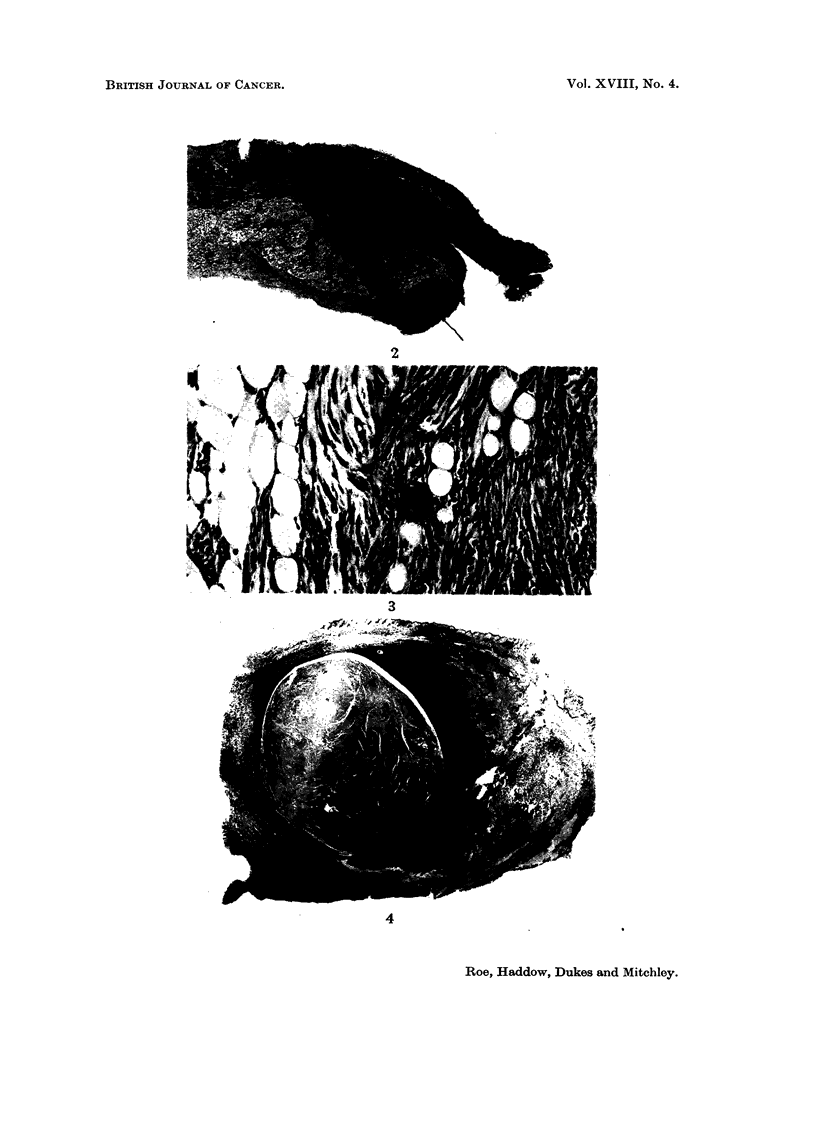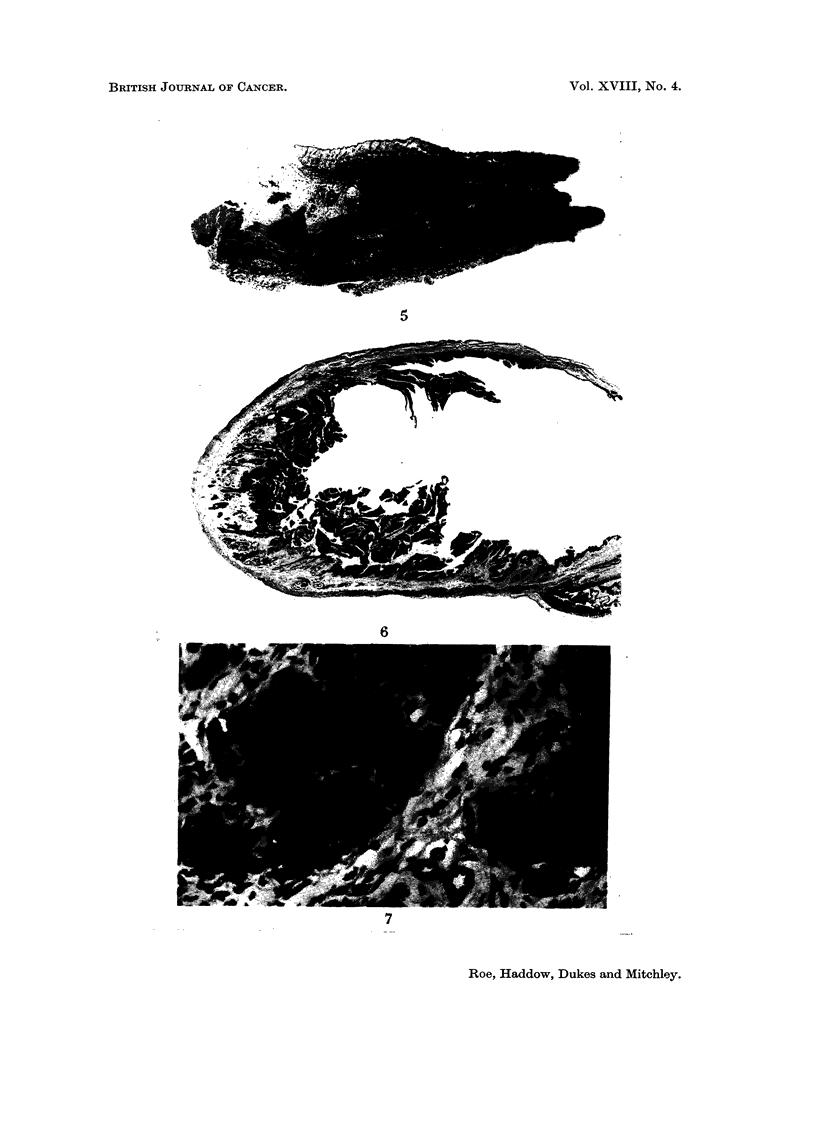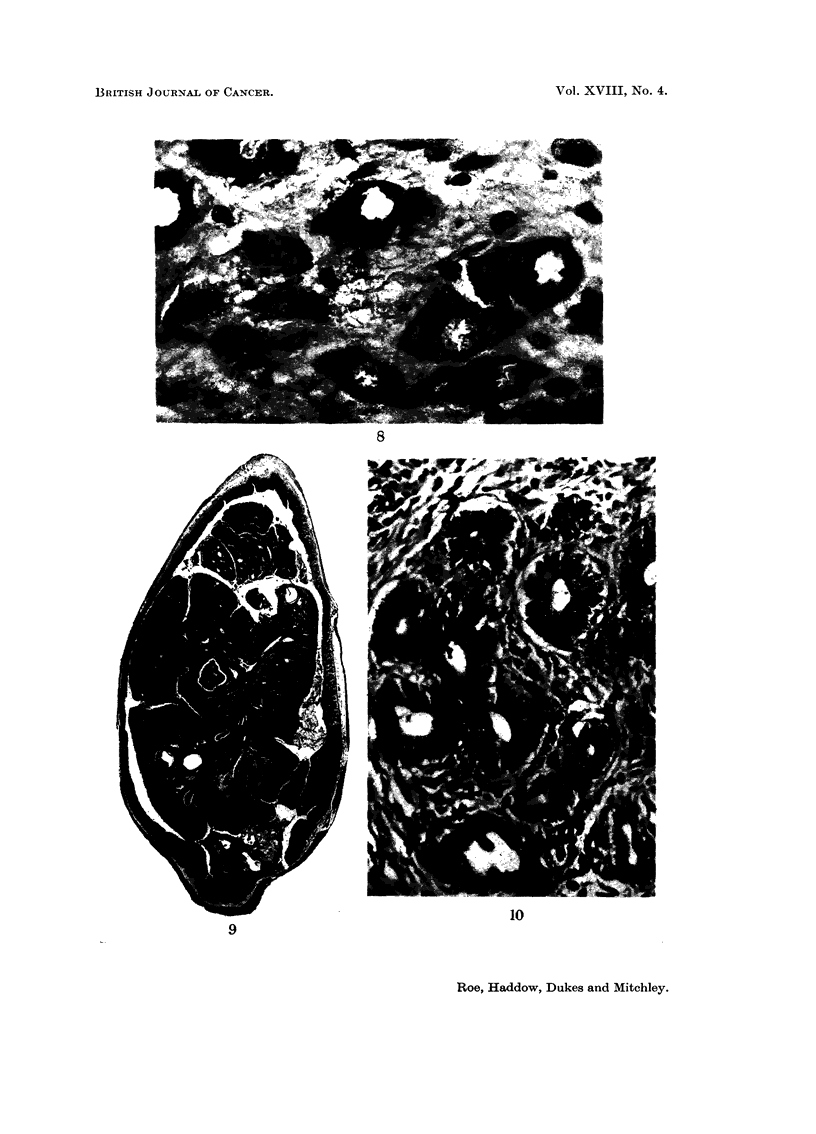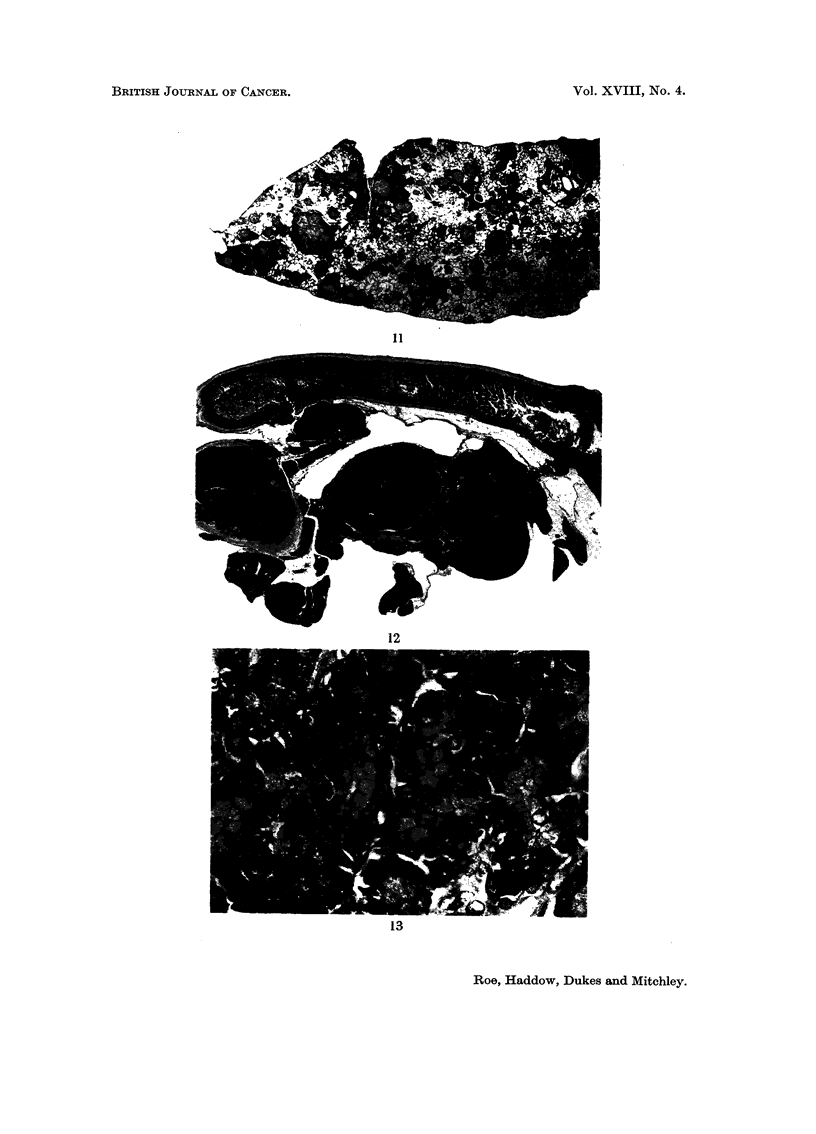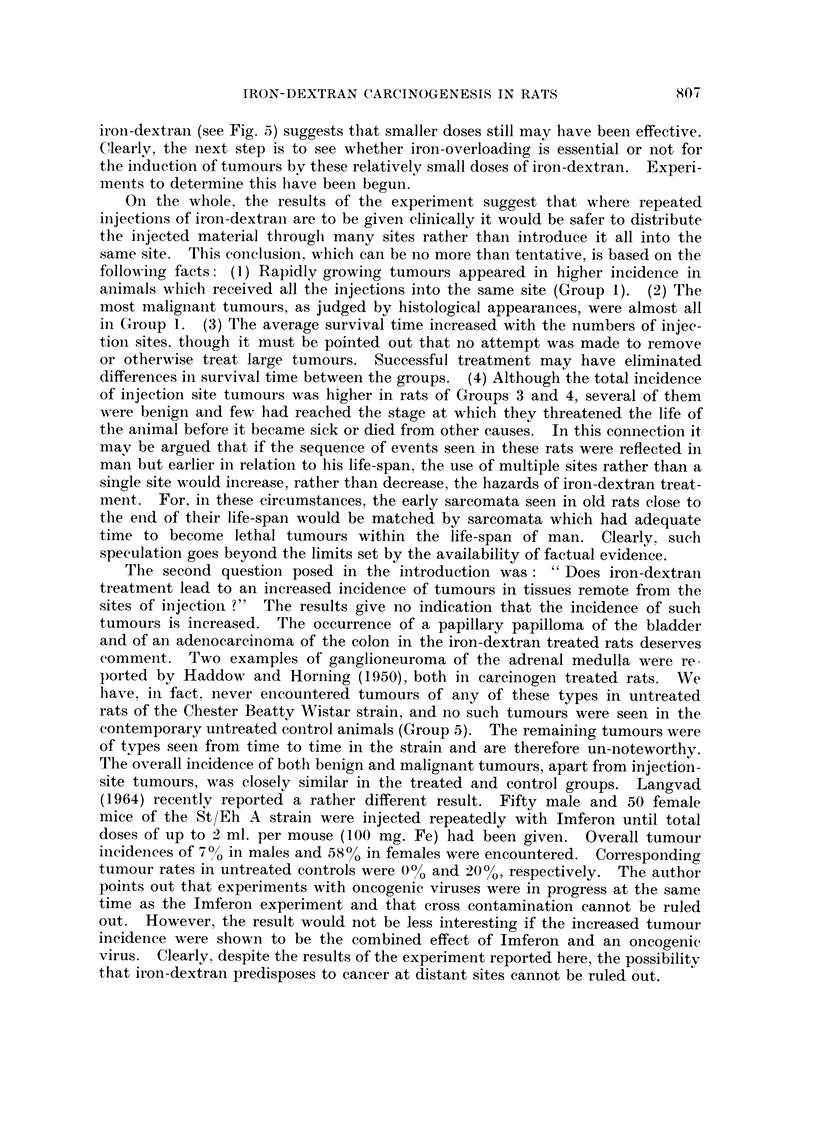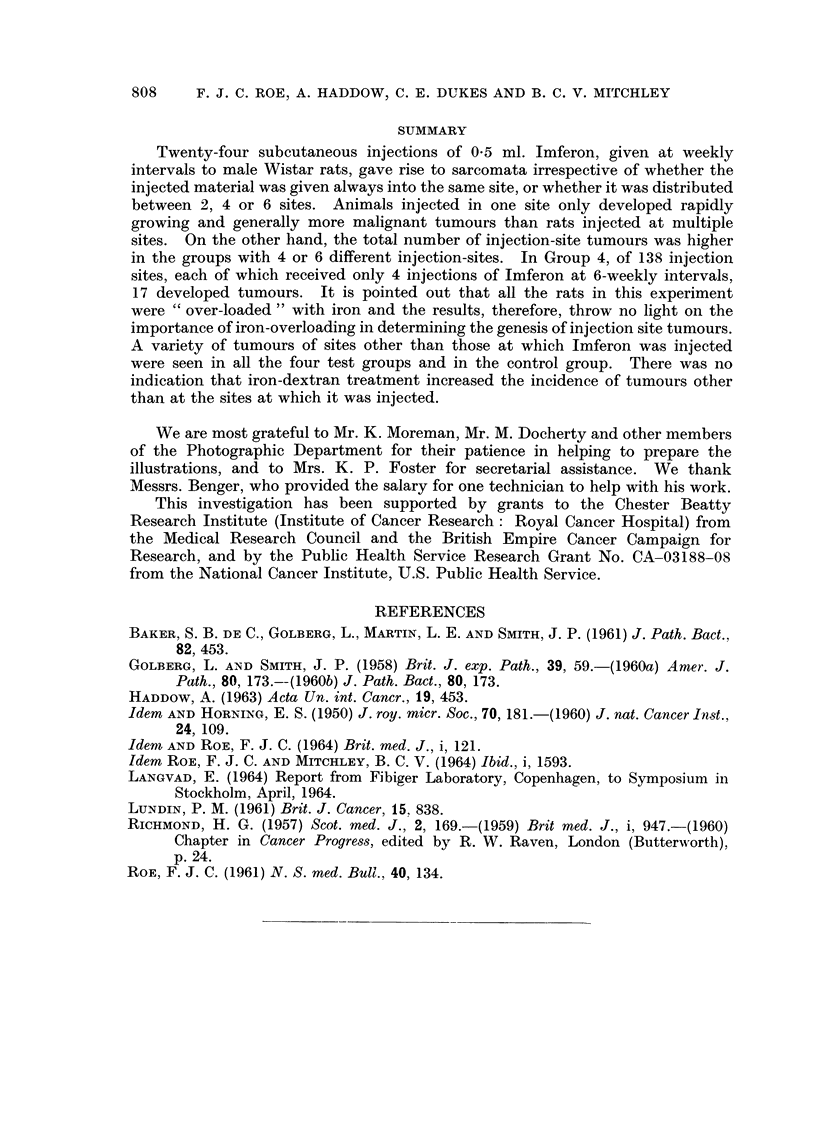# Iron-Dextran Carcinogenesis in Rats: Effect of Distributing Injected Material Between One, Two, Four, or Six Sites

**DOI:** 10.1038/bjc.1964.93

**Published:** 1964-12

**Authors:** F. J. C. Roe, A. Haddow, C. E. Dukes, B. C. V. Mitchley

## Abstract

**Images:**


### IRON-DEXTRAN CARCINOGENESIS IN RATS: EFFECT OF DISTRIBUTING INJECTED MATERIAL BETWEEN ONE, TWO, FOUR, OR SIX SITES.

F. J. C. ROE, A. HADDOW, C. E. DUKES AND B. C. V. MITCHLEY

*From the Chester Beatty Research Institute, Institute of Cancer Research : Royal Cancer Hospital, Fulham Road London, S.W.3*

Received for publication October 1, 1964

It is beyond dispute that when repeated subcutaneous injections of the iron-dextran complex "Imferon" are given to rats, mice, hamsters or rabbits, malignant tumours may arise in high incidence at the injection site (Richmond, 1957, 1959, 1960 ; Haddow and Horning, 1960 ; Lundin, 1961 ; Haddow, Roe and Mitchley, 1964). It has been argued that the animal experiments in which tumours have been induced involved the injection of doses of iron-dextran much larger in relation to body weight than any used clinically ; and that in all the tumour-bearing animals there was also evidence of gross iron overloading (Goldberg and Smith, 1958, 1960*a*, 1960*b* ; Baker, Goldberg, Martin and Smith, 1961). Haddow and Horning (1960) and later Roe (1961) pointed out that since the size of body cells is similar in man and laboratory animals, the actual dose, and not the body weight, may be the critical factor in determining the induction of tumours at the site of injection of a foreign material into the body : a particular volume of material will come into contact with approximately the same number of body cells in the rat as it does in man. On the question of iron-overloading, which according to Baker *et al.* (1961) delays the removal of iron from the injection site, Haddow and Roe (1964), whilst unable to dismiss its importance entirely, pointed out that injection-site tumours may appear in response to doses of iron-dextran much lower than those used in other experiments so far described in the literature.

The purpose of the experiment to be described below was two-fold. The first was to see whether the incidence and time of appearance of tumours in rats given a fixed number of injections of iron-dextran would be affected by distributing the injections between two, four or six sites, instead of giving them, as in previous experiments (Haddow and Horning, 1960), all into the same site. It was hoped that the results of the experiment would indicate whether, in the case of courses of injections of Imferon given clinically, it was better always to inject the material into the same site or to use multiple sites. The second object was to see whether iron-dextran increased the incidence of tumours in tissues distant from the site of injection. In previous experiments a higher than expected incidence of such tumours had been encountered (Haddow, 1963 ; Langvad, 1964).

### MATERIALS AND METHODS

### *Rats*

128 Male rats of the Chester Beatty stock Wistar strain were used for the experiment. At six weeks of age they were divided randomly into 4 groups of 24 and 1 of 32, and thereafter housed in metal cages 8 to a cage. Throughout the experiment they were fed a cubed diet, Diet 86, prepared by Messrs. Dixon & Sons of Ware, Herts. This diet contains whole ground wheat—50%, whole ground barley—25%, white fish meal—7%, meat and bone meal—6%; dried brewer's yeast—5%, dried grass meal—5%, cod liver oil—1%, and salt—1%. Water was given *ad libitum.* Rats of the Chester Beatty strain are especially large and at the age of 6–7 weeks, when the first injection was given, the animals weighed approximately 200 g. each.

*Iron-dextran complex : Imferon*

"Imferon" (Bengers' Laboratories) was used for all injections. This preparation of iron-dextran contains 50 mg. iron per ml.

*Experimental details*

The four groups which contained 24 rats each were treated, from the 7th week of life onwards, with 24 once-weekly subcutaneous injections of 0·5 ml. Imferon. In the case of Group 1 all the injections were into the same site, namely, the right groin. Rats of Group 2 were injected alternately in the right groin and left posterior axillary region, each site being injected once every second week for a total of 12 times. Rats of Group 3 were injected into both groins and both posterior axillary regions in the following rotation: right groin, left posterior axilla, left groin, right posterior axilla. In this case each site was injected every fourth week and only 6 times in all. In the case of Group 6, six sites were used, the two additional ones being in the sacral region near the root of the tail and a point between the anterior angles of the scapulae. Again the sites were injected in rotation, each site receiving 4 injections at 6-week intervals. Fig. 1 shows the positions of the injection sites and indicates the order in which they were injected.

The group which contained 32 rats (Group 5) was left untreated but in all other respects kept under the same conditions as Groups 1–4.

Animals were examined twice each week and killed if they developed rapidly growing or large tumours at any of the injection sites, or if they became sick from any other cause. Occasionally, animals which had not previously been noted as sick were found dead. Except where autolytic changes precluded it, a careful and full post-mortem examination was carried out. Tissue was taken for histological examination from each injection site, irrespective of whether a tumour was thought to be present or not. At the same time sections were prepared from suspected tumours of other sites in the body.

### RESULTS

### *Tumours Arising at the Sites of Injection of Iron-Dextran*

The analysis of the experimental results proved somewhat more difficult than anticipated. As in all long-term experiments of this kind some of the animals died from intercurrent infections before they developed tumours. In addition, two rats of Group 1 and one of Group 2, which were thought to have tumours at the site of injection of iron-dextran, died and autolysis prevented histological assessment. Another problem was that increasing thickening, due to general fibrosis, often preceded the appearance of tumours and the exact time of change from this state to neoplasia was difficult to determine by simple inspection and palpation of the living animal. The tumours which arose at the injection sites were of several different histological varieties. In some cases large tumours proved to be benign fibromata, whereas much smaller ones were early sarcomata, as judged by the usual histological criteria. In the case of animals killed because of a large tumour at one of multiple injection sites, tumours in earlier stages of development were found at some or all of the remaining sites. Sometimes the presence of these additional tumours was suspected whilst the animal was still alive, and sometimes they were discovered for the first time at post-mortem. *Frequently, multiple neoplastic foci were found at a single injection site.* Finally, since only one or two sections prepared from each injection site were examined microscopically, it is possible that early tumours were in some cases missed.

The results, as far as tumours at the sites of injection are concerned, are given in Table I. *The highest incidence of rapidly growing sarcomata at the injection site was encountered in Group* 1. In this group the first undoubted sarcoma appeared after 329 days and the animal had to be killed because of it on the 354th day. Altogether, 14 out of 22 rats of this group (64%) which came to post-mortem developed large sarcomata for which they had to be killed, the average time of killing being 501 days. As stated above, autolysis prevented post-mortem examination in the case of two rats ; both of these were considered on the basis of macroscopic examination to have sarcomata, but have been excluded from the results shown in Table I. No sarcomata of microscopic dimensions and no benign tumours were found at the injection site in any of the remaining rats of this group. The lowest incidence of rapidly growing sarcomata for which rats had to be killed was encountered in Group 4 : only 3 out of 23 rats (13%) developed such tumours and these were killed after an average of 574 days. However, when injection sites showing sarcomata of microscopic dimensions were taken into account the

TABLE I.—*Tumours at the Sites of Inspection of Iron-Dextran*

| Group | 1 | 2 | 3 | 4 | 5 |
|---|---|---|---|---|---|
| Number of rats | 24 | 24 | 24 | 24 | 32 |
| Number examined post-mortem | 22 | 23 | 24 | 23 | 28 |
| Number of injection sites per rat | 1 | 2 | 4 | 6 | 0 |
| Number of rats killed because of a histologically malignant tumour at one or more injection sites | 14 | 6 | 9 | 3 | — |
| Average time at which rats were killed for such tumours (days) | 501 | 667 | 706 | 674 | — |
| Time of appearance of first malignant tumour in group (days) | 329 | 460 | 449 | 522 | — |
| Total number of rats which developed malignant tumours at one or more injection site | 14 | 6 | 9 | 7 | — |
| Total number of rats which developed benign or malignant tumours at one or more injection site | 14 | 7 | 12 | 10 | — |
| Number of rats dying without a tumour at any injection site | 8 | 16 | 12 | 13 | 28 |
| Average survival of all rats in Group (days) | 505 | 539 | 582 | 608 | 573* |
| Average survival of rats examined post-mortem (days) | 514 | 541 | 582 | 622 | 596* |
| Proportion of injection sites examined post-mortem showing tumour formation | 14/22 (63·6%) | 11/46 (23·9%) | 29/96 (30·2%) | 17/138 (12·3%) | — |

* N.B.—Five apparently healthy control rats were killed on the 732nd day of the experiment after all the rats in the 4 other groups had died or been killed because they were sick. If these animals had been permitted to live out their life-span, the average survival of the controls would have been close to that of Group 4.

incidence was 7 out of 23 (30%); and when, in addition, rats bearing benign tumours at the injection site were also included, the total incidence of animals with injection-site tumours became 10 out of 23 (43%). It was not uncommon for multiple sites to be involved simultaneously by tumour formation. Another way, therefore, of assessing the results in Groups 1 and 4 is to compare the ratio of the numbers of tumours to the number of sites injected with iron-dextran (see final column of Table I). It may be seen that in Group 1, 14 out of the 22 sites examined post-mortem developed tumours, i.e. 64% of the sites injected. In Group 4, 17 out of the 138 sites examined (i.e. 12·3%) had tumours. The 22 sites in Group 1 each received 6 times as many injections as the 138 sites in Group 4. Thus, the incidence of tumours at the injection site in the two groups was approximately proportional to the number of injections and to the total dose of iron-dextran introduced into the site.

In general, the results in Groups 2 and 3 were intermediate between those in Groups 1 and 4 with regard to (1) the incidence of large sarcomata for which rats had to be killed, (2) the time of appearance of the first sarcoma, (3) the average time of killing because of large sarcomata, (4) the incidence of microscopic sarcomata and of benign tumours at the injection site, and (5) the ratio of tumourous sites to sites injected. However, in some respects the tumorigenic response in Group 2 was less impressive than that in Group 3, which was surprising. In Group 3 for instance, tumours developed in 29 out of 96 sites examined (30·2%), whereas in Group 2, the corresponding figures were 11 out of 46 sites (23·9%).

It is undoubtedly difficult to obtain a clear picture of the results of the experiment. One of the main reasons for this arises from the difficulty of interpreting the lesions which were regarded by us as early sarcomata. It cannot, in our view, be seriously doubted that these were truly malignant in nature, but it is difficult to judge how quickly they would have become large and obvious tumours if the animals bearing them had been allowed to live longer.

Perhaps the most helpful way of looking at the results is to compare average survival (see Table I) and the incidence of animals which developed no injection-site tumours in the various groups. Only 8 rats of Group 1, which were examined post-mortem, had no tumour at the injection site. The comparable figures for Groups 2, 3 and 4, in which multiple injection sites were available for tumour development, were 16, 12 and 13, respectively. Using this method of assessment of the results it would seem to be advisable, where multiple injections of iron-dextran are to be given, to use multiple rather than just a single site. Similarly, a comparison of the average survival in the 5 groups indicates a progressive increase with increasing numbers of sites used: the averages for Groups 1–4 were 505, 539, 582 and 608 days, respectively. As explained in the footnote to Table I, 5 quite healthy control rats were killed off on the 732nd day (i.e. shortly after the last of the iron-dextran treated rats had had to be killed because of tumour development or because it was sick). Had these five rats been allowed to live out their life span the average survival of the control group would have been close to that of Group 4.

*Pathology of tumours arising at sites of injection of iron-dextran*

The earliest change discernible at the injection site on gross examination was a palpable thickening. This was associated with the microscopic appearance of fibrosis in relation to collections of macrophages containing iron pigment. From such fibrotic areas large tumours arose, sometimes suddenly, sometimes insidiously. The more rapidly growing of these large tumours proved on histological examination to be fibrosarcomata or pleomorphic sarcomata. Some of the more slowly growing tumours were sarcomata and some benign fibromata. There was a marked difference between Group 1 and the other groups in that no benign tumours were seen in the former. Moreover, the most pleomorphic and mitotically active tumours were seen in this group.

Sarcomata of microscopic size (Fig. 2 and 3) were seen at one or more injection sites in Group 2 (2 cases), Group 3 (6 cases) and Group 4 (5 cases). Multiple neoplastic lesions of the same type, or of different types, were often seen in a single injection site (Fig. 4 and 5).

Apart from sarcomata and fibromata, two small adenomatous lesions were seen. Presumably these arose from mammary gland elements, though all the rats used in the experiment were males.

TABLE II.—*Tumours Arising Remotely from the Injection-sites in Iron-Dextran Treated Rats*

| Group | Number of rats examined post-mortem | Number of rats with benign or malignant tumours other than at injection sites | Details of benign tumours No. | Details of benign tumours Description | Details of malignant tumours No. | Details of malignant tumours Description |
|---|---|---|---|---|---|---|
| 1 | 22 | 4 | 3 | Papilloma of bladder (Fig. 6 and 7)<br>Mammary fibroadenoma<br>Ganglioneuroma (Fig. 8) | 1 | Lymphoma. |
| 2 | 23 | 2 | 1 | Mammary fibroma | 1 | Myxosarcoma of lip. |
| 3 | 24 | 3 | 1 | Polypoid adenoma of colon (Fig. 9 and 10) | 2 | Sarcoma of lung.<br>Lymphoma. |
| 4 | 23 | 3 | 0 | | 3 | Abdominal sarcoma.<br>Osteosarcoma (Fig. 11).<br>Lymphoma. |
| | 92 | 12 (13·0%) | 5 | | 7 (7·6%) | |
| 5 (Controls) | 28 | 3 (10·7%) | 1 | Multiple adenomata (exocrine) of pancreas (Fig. 12 and 13). | 2 (7·1%) | Lymphoma.<br>Abdominal sarcoma. |

### *Tumours Arising Remotely from the Sites of Injection of Iron-Dextran*

Details of tumours arising remotely from the injection sites are given in Table II. An interesting variety of tumours, some benign and some malignant, were seen. The overall incidence of both benign and malignant tumours in the iron-dextran treated rats (Groups 1–4) was closely similar to that in the untreated controls (Group 5). The data are insufficient for comment regarding possible differences in the types of tumour arising in treated and control animals. (Some of the tumours are illustrated in Fig. 6–13).

### DISCUSSION

The fact that benign or malignant tumours arose at 71 out of 302 injection sites in 92 rats treated with iron-dextran indicates once again that the carcinogenicity of this material given in this way is of no low order. On the other hand, the results of the experiment throw no light on the importance of the presence of iron-overloading in the genesis of tumours at the site of injection of iron-dextran (Golberg and Smith, 1958, 1960*a*, 1960*b*; Baker, Golberg, Martin and Smith, 1961). Each rat in Groups 1–4 received a total of 12 ml. of iron-dextran (600 mg. iron). On the basis of comparative body weight, this treatment would be the equivalent of the introduction of over 2 litres of iron-dextran into a non-anaemic 70 kg. man. It is not certain from the present experiment that any injection-site tumours would have arisen if the rats had not been overloaded with iron. On the other hand, it is quite clear that the introduction of quite small total doses of iron-dextran into a single injection site (in an iron-overloaded rat) is capable of giving rise to tumours. Each injection site in the case of rats of Group 4 of the present experiment received only 4 injections of 0·5 ml. iron-dextran, a total of 2 ml., and the risk of a benign or malignant tumour arising at such a site was as high as 12·3% (the risk of a malignant tumour developing was 8·7%). The fact that multiple malignant tumours were sometimes seen in sites exposed to only 2 ml. of

---

### EXPLANATION OF PLATES

FIG. 1.—Positions of the six injection sites.

FIG. 2.—Subcutaneous injection site from left groin of rat of Group 4. This site received four injections of 0·5 ml. iron-dextran at six-weekly intervals, the last injection being given approximately 50 weeks before the animal was killed because of the development of large sarcomata at each of 2 other injection sites. Large deposits of siderophages lie in the dermis and subcutaneous tissues and a rounded early sarcomatous lesion can be seen in the deepest part of the section. H. & E. ×5.

FIG. 3.—Edge of early sarcoma from the left posterior axillary region of a rat of Group 4 killed 2 years after the start of treatment. The lesion extends into fatty tissue and consists of fibroblasts and fibrocytes. Some of the cells near the edge of the lesion show a myxomatous change. This was a frequent finding in such lesions. H. & E. ×120.

FIG. 4.—Encapsulated fibroma partly surrounded by actively growing fibro-sarcoma from left axilla of a rat of Group 3 killed 2 years after the start of the experiment. As in other cases, the tumours have arisen in close proximity to deposits of iron-laden macrophages. H. & E. ×3.

FIG. 5.—Multiple early sarcomata at the site of injection of Imferon in right axilla of the same rat as depicted in Fig. 2. At least 5 distinct foci are visible in this section. H. & E. ×3.

FIG. 6.—Multiple benign papillomata involving half the circumference of the bladder of a rat of Group 1. This animal was killed during the 47th week of the experiment because of a large sarcoma at the site of injection. The bladder lesion was an incidental finding. H. & E. × 4·5.

FIG. 7.—Same as Fig. 6, to show well-differentiated nature of transitional epithelium of which the tumour consists. H. & E. ×255.

FIG. 8.—Ganglioneuroma of the adrenal medulla from a rat of Group 1, which was killed with a large injection-site sarcoma during the 94th week of the experiment. H. & E. ×560.

FIG. 9.—Polypoid adenoma of colon from rat of Group 3 killed because of tumours at 3 of four injection sites 2 years after the start of treatment. The tumour is causing considerable distension of the gut. H. & E. ×7.

FIG. 10.—Same as Fig. 9 showing the well-differentiated acinar structure of the tumour. H. & E. ×255.

FIG. 11.—Multiple deposits of osteogenic sarcoma in the lung of an untreated rat of Group 5. The animal was killed because it became sick during the 90th week of the experiment. Similar deposits were present in several other organs. The site of the primary lesion was not ascertained. H. & E. ×3·5.

FIG. 12.—Adenomatosis of the pancreas of an untreated rat of Group 5 killed at the termination of the experiment during the 105th week. The pancreas is seen attached to a loop of small gut. Three large and multiple smaller adenomatous nodules may be seen. H. & E. ×3·5.

FIG. 13.—Same as Fig. 12 showing the well-differentiated acinar structure of one of the nodules and a prominent mitotic figure in one of the cells. H. & E. ×515.

1

2

3

4

Roe, Haddow, Dukes and Mitchley.

5

6

7

Roe, Haddow, Dukes and Mitchley.

8

9

10

Roe, Haddow, Dukes and Mitchley.

11

12

13

iron-dextran (see Fig. 5) suggests that smaller doses still may have been effective. Clearly, the next step is to see whether iron-overloading is essential or not for the induction of tumours by these relatively small doses of iron-dextran. Experiments to determine this have been begun.

On the whole, the results of the experiment suggest that where repeated injections of iron-dextran are to be given clinically it would be safer to distribute the injected material through many sites rather than introduce it all into the same site. This conclusion, which can be no more than tentative, is based on the following facts: (1) Rapidly growing tumours appeared in higher incidence in animals which received all the injections into the same site (Group 1). (2) The most malignant tumours, as judged by histological appearances, were almost all in Group 1. (3) The average survival time increased with the numbers of injection sites, though it must be pointed out that no attempt was made to remove or otherwise treat large tumours. Successful treatment may have eliminated differences in survival time between the groups. (4) Although the total incidence of injection site tumours was higher in rats of Groups 3 and 4, several of them were benign and few had reached the stage at which they threatened the life of the animal before it became sick or died from other causes. In this connection it may be argued that if the sequence of events seen in these rats were reflected in man but earlier in relation to his life-span, the use of multiple sites rather than a single site would increase, rather than decrease, the hazards of iron-dextran treatment. For, in these circumstances, the early sarcomata seen in old rats close to the end of their life-span would be matched by sarcomata which had adequate time to become lethal tumours within the life-span of man. Clearly, such speculation goes beyond the limits set by the availability of factual evidence.

The second question posed in the introduction was : " Does iron-dextran treatment lead to an increased incidence of tumours in tissues remote from the sites of injection ?" The results give no indication that the incidence of such tumours is increased. The occurrence of a papillary papilloma of the bladder and of an adenocarcinoma of the colon in the iron-dextran treated rats deserves comment. Two examples of ganglioneuroma of the adrenal medulla were reported by Haddow and Horning (1950), both in carcinogen treated rats. We have, in fact, never encountered tumours of any of these types in untreated rats of the Chester Beatty Wistar strain, and no such tumours were seen in the contemporary untreated control animals (Group 5). The remaining tumours were of types seen from time to time in the strain and are therefore un-noteworthy. The overall incidence of both benign and malignant tumours, apart from injection-site tumours, was closely similar in the treated and control groups. Langvad (1964) recently reported a rather different result. Fifty male and 50 female mice of the St/Eh A strain were injected repeatedly with Imferon until total doses of up to 2 ml. per mouse (100 mg. Fe) had been given. Overall tumour incidences of 7% in males and 58% in females were encountered. Corresponding tumour rates in untreated controls were 0% and 20%, respectively. The author points out that experiments with oncogenic viruses were in progress at the same time as the Imferon experiment and that cross contamination cannot be ruled out. However, the result would not be less interesting if the increased tumour incidence were shown to be the combined effect of Imferon and an oncogenic virus. Clearly, despite the results of the experiment reported here, the possibility that iron-dextran predisposes to cancer at distant sites cannot be ruled out.

SUMMARY

Twenty-four subcutaneous injections of 0·5 ml. Imferon, given at weekly intervals to male Wistar rats, gave rise to sarcomata irrespective of whether the injected material was given always into the same site, or whether it was distributed between 2, 4 or 6 sites. Animals injected in one site only developed rapidly growing and generally more malignant tumours than rats injected at multiple sites. On the other hand, the total number of injection-site tumours was higher in the groups with 4 or 6 different injection-sites. In Group 4, of 138 injection sites, each of which received only 4 injections of Imferon at 6-weekly intervals, 17 developed tumours. It is pointed out that all the rats in this experiment were " over-loaded " with iron and the results, therefore, throw no light on the importance of iron-overloading in determining the genesis of injection site tumours. A variety of tumours of sites other than those at which Imferon was injected were seen in all the four test groups and in the control group. There was no indication that iron-dextran treatment increased the incidence of tumours other than at the sites at which it was injected.

We are most grateful to Mr. K. Moreman, Mr. M. Docherty and other members of the Photographic Department for their patience in helping to prepare the illustrations, and to Mrs. K. P. Foster for secretarial assistance. We thank Messrs. Benger, who provided the salary for one technician to help with his work.

This investigation has been supported by grants to the Chester Beatty Research Institute (Institute of Cancer Research : Royal Cancer Hospital) from the Medical Research Council and the British Empire Cancer Campaign for Research, and by the Public Health Service Research Grant No. CA–03188–08 from the National Cancer Institute, U.S. Public Health Service.

REFERENCES

Baker, S. B. de C., Golberg, L., Martin, L. E. and Smith, J. P. (1961) *J. Path. Bact.*, **82**, 453.

Golberg, L. and Smith, J. P. (1958) *Brit. J. exp. Path.*, **39**, 59.—(1960*a*) *Amer. J. Path.*, **80**, 173.—(1960*b*) *J. Path. Bact.*, **80**, 173.

Haddow, A. (1963) *Acta Un. int. Cancr.*, **19**, 453.

*Idem* and Horning, E. S. (1950) *J. roy. micr. Soc.*, **70**, 181.—(1960) *J. nat. Cancer Inst.*, **24**, 109.

*Idem* and Roe, F. J. C. (1964) *Brit. med. J.*, i, 121.

*Idem* Roe, F. J. C. and Mitchley, B. C. V. (1964) *Ibid.*, i, 1593.

Langvad, E. (1964) Report from Fibiger Laboratory, Copenhagen, to Symposium in Stockholm, April, 1964.

Lundin, P. M. (1961) *Brit. J. Cancer*, **15**, 838.

Richmond, H. G. (1957) *Scot. med. J.*, **2**, 169.—(1959) *Brit med. J.*, i, 947.—(1960) Chapter in *Cancer Progress*, edited by R. W. Raven, London (Butterworth), p. 24.

Roe, F. J. C. (1961) *N. S. med. Bull.*, **40**, 134.